# Autophagy-Dependent Secretion: Crosstalk between Autophagy and Exosome Biogenesis

**DOI:** 10.3390/cimb46030142

**Published:** 2024-03-08

**Authors:** Ekaterina Zubkova, Alexander Kalinin, Anastasya Bolotskaya, Irina Beloglazova, Mikhail Menshikov

**Affiliations:** 1 National Medical Research Centre of Cardiology Named after Academician E.I. Chazov, 121552 Moscow, Russia; 2 Faculty of Fundamental Medicine, Lomonosov Moscow State University, 119991 Moscow, Russia; 3 Institute of Clinical Medicine, Sechenov University, 119435 Moscow, Russia

**Keywords:** secretory authophagy, exosomes, amphisomes, stress, unconventional protein secretion

## Abstract

The cellular secretome is pivotal in mediating intercellular communication and coordinating responses to stressors. Exosomes, initially recognized for their role in waste disposal, have now emerged as key intercellular messengers with significant therapeutic and diagnostic potential. Similarly, autophagy has transcended its traditional role as a waste removal mechanism, emerging as a regulator of intracellular communication pathways and a contributor to a unique autophagy-dependent secretome. Secretory authophagy, initiated by various stress stimuli, prompts the selective release of proteins implicated in inflammation, including leaderless proteins that bypass the conventional endoplasmic reticulum–Golgi secretory pathway. This reflects the significant impact of stress-induced autophagy on cellular secretion profiles, including the modulation of exosome release. The convergence of exosome biogenesis and autophagy is exemplified by the formation of amphisomes, vesicles that integrate autophagic and endosomal pathways, indicating their synergistic interplay. Regulatory proteins common to both pathways, particularly mTORC1, emerge as potential therapeutic targets to alter cellular secretion profiles involved in various diseases. This review explores the dynamic interplay between autophagy and exosome formation, highlighting the potential to influence the secretome composition. While the modulation of exosome secretion and cytokine preconditioning is well-established in regenerative medicine, the strategic manipulation of autophagy is still underexplored, presenting a promising but uncharted therapeutic landscape.

## 1. Introduction

Extracellular vesicles (EVs) have emerged as vital intercellular messengers, contributing to the complex landscape of cellular signaling. As an integral part of normal cellular function, almost all cell types continuously release a variety of vesicles into the extracellular milieu. EVs encapsulate various biological molecules, such as proteins, lipids, and a range of RNA species, including mRNAs, long noncoding RNAs, and microRNAs (miRNAs) [1]. The classification of these EVs is based on their size and biogenesis characteristics, comprising three main subtypes: microvesicles (MVs), exosomes, and apoptotic bodies, in addition to specific types like exomers, oncosomes, and mygrasomes. The diversity of EVs is continuously expanding and now includes oncosomes, large oncosomes, enveloped viruses, exomeres, exophers, migrasomes, supramolecular attack particles, and elongated particles.

Among the secreted microvesicles, exosomes—bilayer vesicular nanoparticles ranging in size from 30 to 150 nm—are a particularly fascinating subset of these secreted vesicles. They contain various cargos, including proteins, mRNAs, and miRNAs [2,3], and have been identified as having significant potential for therapeutic and diagnostic applications [4]. Exosomes are released from cells through a specific, multi-step exocytotic pathway [5]. Their genesis involves budding into endosomes to form multivesicular bodies (MVBs) within the cytoplasm. These MVBs eventually merge with the cell membrane, leading to the release of exosomes into the extracellular milieu.

Initially, it was believed that exosomes served merely as a cellular waste disposal system, packaging and secreting unwanted cellular debris into the extracellular environment. However, while this may still be partly correct, evolving research has illuminated their role in cell-to-cell communication, acting as effectors in physiological and pathological processes and serving as disease biomarkers [6].

In parallel to exosome biology, autophagy is a cellular process integral to the maintenance and regulation of the intracellular environment. This metabolic pathway targets the degradation of cell constituents, clearing out dysfunctional or unnecessary components through a lysosome-dependent mechanism. Autophagy is not limited to waste disposal; it is also intricately involved in unconventional protein secretion (UPS) pathways, which are fundamental for disposing of toxic proteins, immune signaling, and pathogen surveillance. Moreover, autophagy encompasses a spectrum of vital functions that participate in intracellular signaling regulation and the replenishment of cellular energy resources, demonstrating its multifaceted nature [7]. The formation of autophagosomes, a pivotal step in recycling cellular contents, shares similarities with the biogenesis of exosomes.

Given their biogenetic parallels and functional roles, it seems likely that exosomes and autophagosomes have a complementary existence within the cell. Supporting this concept is the presence of amphisomes, which blend the characteristics and functionalities of both vesicle types and contribute to cellular secretion machinery [8].

Secretory autophagy, a process wherein autophagosomes or related structures fuse with the cell membrane to release their contents, underscores the complexity of cellular secretion. This mechanism enables the export of cytosolic cargo, such as certain cytosolic proteins without an N-terminal leader peptide (e.g., Interleukin 1β (IL-1β) and IL-18), circumventing the conventional secretory pathway associated with the endoplasmic reticulum and Golgi apparatus [9]. Thus, secretory autophagy is more than just waste removal; it facilitates the release of specific signaling molecules [10]. The strategic decision to direct materials either to lysosomal degradation or to extracellular secretion is a critical decision for the autophagic machinery, with far-reaching implications for both health and disease.

Understanding the autophagic machinery is pivotal, as we hypothesize the existence of an autophagy-dependent secretome in vivo—a concept that requires exploration and a deeper understanding of the autophagic molecular machinery. Consequently, it has become evident that the expanding body of research on the interplay between secretory autophagy and exosome release offers invaluable insight into this area of cell biology.

## 2. Autophagy Machinery

Autophagy is a multifaceted cellular process, primarily characterized by its role in degrading and recycling cytoplasmic components through a lysosome-mediated mechanism. It can be categorized into several forms including macroautophagy, the most common form, where cytoplasmic content is sequestered into double-membrane vesicles known as autophagosomes and delivered to lysosomes for breakdown and recycling. In microautophagy, the lysosomal membrane directly engulfs material, whereas chaperone-mediated autophagy selectively targets specific proteins for lysosomal translocation and breakdown. Mitophagy, another form, focuses on the removal of damaged mitochondria, thus preserving cellular energy management and integrity. Additionally, there are more specialized forms, such as pexophagy, for degrading peroxisomes and others that target specific cellular components [9].

Apart from these degradative functions, autophagy also engages in non-degradative processes, such as secretory autophagy. This unique pathway diverges from the conventional route, enabling cells to release proteins and other substances into the extracellular environment instead of directing them towards lysosomal degradation. This selective secretion extends autophagy’s role beyond intracellular turnover to include significant involvement in cellular secretion and distribution of materials [11]. The autophagic machinery’s integration with these processes underscores its essential contribution to cellular organization and function, highlighting a complex network that transcends simple cellular cleanup.

All types of autophagy can be induced by various stimuli such as nutrient starvation, lack of growth factors, stress, immune signals, or cellular demands. Autophagy involves several major stages: initiation, nucleation (budding), elongation and maturation, fusion and degradation [12]. A key regulator of these events is the mechanistic target of rapamycin (mTOR) kinase located within the mTORC1 complex. In the active state, mTORC1 suppresses autophagy (mainly through phosphorylation of ULK1 kinase), while its negative regulation (AMP-activated protein kinase (AMPK), P53 signaling and several others) activates it.

unc-51, like autophagy activating kinase 1 (ULK1) complex, consisting of ULK1 kinase, focal adhesion kinase family interacting protein of 200 kD (FIP200), and autophagy-related protein 13 (Atg13), is activated under stress conditions [7]. mTORC1 negatively regulates autophagy by inhibiting the activity of the Atg1 (ULK1) complex via direct phosphorylation. When mTORC1 is inactive, ULK1 is dephosphorylated, resulting in autophagy activation [13].

Beclin-1, an important molecule participating in autophagosome formation at the nucleation stage, forms a complex with factors PI3 kinase type III (VPS34) and Atg14L, promoting the generation of phosphorylated phosphatidylinositol (PtdIns) and recruiting proteins from the cytoplasm [14,15].

During the elongation and maturation stages, two ubiquitin-like conjugation systems are required for autophagosome membrane extension. The first one involves conjugation to phosphatidylethanolamine (PE) of microtubule-associated light chain protein 3 (LC3). LC3 is cleaved by Atg4 at its C-terminus to form intracellular LC3-I, which conjugates to PE in Atg7 and Atg3 ubiquitin-like reactions. This lipid form of LC3 (LC3-II) then attaches to the autophagosome membrane [16]. The second system involves the Atg12-Atg5-Atg16 complex, in which Atg12 is conjugated to Atg5 via the ubiquitin-like reactions of Atg7 and Atg10 [17]. The Atg12-Atg5 conjugate interacts non-covalently with Atg16 to form a large complex [18]. Once autophagosomes are formed, they can either fuse with lysosomes to form autolysosomes or with organelles of endosomal origin, such as late endosomes, to form amphisomes.

Of note, secretory autophagosomes are distinct both qualitatively and functionally from their degradative counterparts, being decorated with Rab8A or Rab8B proteins, respectively. However, both types are LC3 positive [19,20].

Secretory autophagy was first identified through research aimed at investigating the extracellular release mechanisms of leaderless proteins, which lack an N-terminal signal sequence and are therefore incapable of entering the endoplasmic reticulum (ER) [21,22]. The pathway, known as Unconventional Protein Secretion (UPS), allows for the secretion of such proteins without the traditional ER-Golgi route [22]. Interleukin 1β is one of the first proteins reported to be secreted extracellularly through this alternative route. Currently, the term ‘secretory autophagy’ encompasses all extracellular secretion processes facilitated by autophagy-related proteins and autophagosomes. This includes both conventional and unconventional protein secretion, extracellular vesicle (EV) production, and the discharge of secretory lysosomes [23].

The process of secretory autophagy can be conceptually divided into three primary stages: (1) cargo recruitment to the forming autophagosome, for instance, through cargo receptors like TRIM16 (tripartite motif-containing protein16); (2) transport of the receptor–cargo complex to the autophagosome membrane via the R-SNARE (SNAP receptor) protein SEC22B; (3) internalization of the cargo protein into the autophagosome, followed by fusion with the plasma membrane [24]. This fusion is facilitated by a combination of SNARE proteins, specifically Sec22 on the autophagosomes, SNAP23 and SNAP29, and their cognate partners, STX (syntaxin)3 and STX4, on the plasma membrane. This ultimately leads to the secretion of cargo proteins into the extracellular milieu.

Secretory autophagy, like other forms of autophagy, is modulated by the mTORC1 complex. It has been shown that mTORC1 directly phosphorylates the Golgi reassembly stacking protein GRASP55 (GORASP2), which is pivotal in the secretion process. GRASP55 orchestrates secretion by tethering autophagosome membranes to lysosomes, aiding autophagosome maturation. Phosphorylated GRASP55 is localized exclusively to the Golgi, where it colocalizes with mTOR. Conversely, inhibition of mTORC1 triggers GRASP55 dephosphorylation and its subsequent relocation to autophagosomes and MVBs, thus promoting protein secretion [25].

The key differences between secretory autophagy and macroautophagy lie in the mechanisms of cargo selection and membrane fusion. Secretory autophagy necessitates a specialized mechanism to facilitate the fusion of autophagosomes with the plasma membrane, enabling the release of their contents outside the cell. These processes are essential for the distinct functional outcomes of secretory autophagy compared to the degradative pathway of macroautophagy.

The selection of cargo for secretion may involve autophagy-related proteins (Atg), such as the ATG8-family members, which in humans include LC3A, LC3B, LC3B2, LC3C, GABARAP (gamma-aminobutyric acis receptor associated protein), GABARAPL1, and GABARAPL2. Notably, the secretion of IL-1β is dependent on ATGs. The association of LC3 with secretory autophagosomes highlights the significant role of the ATG8 family in cellular secretion. However, since many secretory autophagy cargoes do not bind to ATG8-family members, it implies that cargo recruitment to secretory autophagosomes may occur through auxiliary mechanisms that are independent of ATG8 proteins. Although ATG8 proteins may only target a restricted set of proteins for secretory autophagy, their primary functions in secretion likely pertain to their essential role in the biogenesis, trafficking, and fusion of autophagosomes [26].

Another mechanism of cargo selection involves the transmembrane emp24 domain-containing protein 10 (TMED10). The C-terminal region of TMED10 interacts with specific motifs in cargo proteins. This interaction is known to trigger the oligomerization of TMED10, which is then likely to form a protein channel within the membrane of the endoplasmic reticulum–Golgi intermediate compartment (ERGIC). Through this channel, leaderless cargoes are translocated into the vesicle carrier for subsequent delivery outside the cell [27].

The TRIM protein family, encompassing approximately 80 members, also serves as cargo receptors in autophagy [28]. Research by Kimura et al. identified several TRIM proteins involved in IL1β secretion, with mature IL1B specifically binding to the TRIM16/ERBBP complex and being directed to the autophagic sequestration membrane. The TRIM16-dependent cargo is subsequently targeted for secretion through fusion with the plasma membrane, and not for degradation by the action of a combination of SNARE proteins, i.e., the R-SNARE Sec22 on autophagosomes and SNAP23, SNAP29, and their associated partners, STX3 and STX4, on the plasma membrane. Sec22b, which regulates ER–Golgi protein trafficking, is of particular importance to autophagy [29].

The stress-responsive protein FKBP51 (the immunophilin FK506 binding protein 51) is a key component and essential driver of the final step of secretory autophagy. FKBP51 functions by chaperoning the hetero-complex assembly of the vesicular R-SNARE SEC22B with the membranous Q-SNAREs, thereby enabling vesicle–membrane fusion [30].

Recent studies have expanded the understanding of secretory autophagy. Tan and colleagues describe a process that they termed Autophagic Secretion of Mitochondria (ASM), where mitochondria can be extracellularly released via a secretory autophagy pathway, even with defects in the mATG8-conjugation machinery, circumventing the lysosomal degradation pathway. This process is crucial for the elimination of dysfunctional mitochondria and is a part of mitochondrial quality control. Functionally, increased ASM promotes the activation of the innate immune cGAS (cyclic GMP-AMP synthase)-STING (stimulator of interferon genes) pathway in recipient cells [31].

Interestingly, STING itself could be secreted via a new organelle, Rafeesome (RAB22A-mediated non-canonical autophagosome fused with an early endosome). Gao and colleagues found that Rab22 small GTPase mediates formation of a non-canonical autophagosome, and activated STING is packaged into these autophagosomes. This non-canonical autophagosome fuses with RAB22A-positive early endosome to become Rafeesome. The Rafeesome, upon formation, evades fusion with lysosomes due to RAB22A’s inhibition of RAB7. Consequently, vesicles containing activated STING are secreted as part of a new type of extracellular vesicle named RAB22A-induced EV (R-EV). These R-EVs play a role in the intercellular transfer of activated STING, promoting antitumor immunity in recipient cells [32]. 

**Figure 1:**
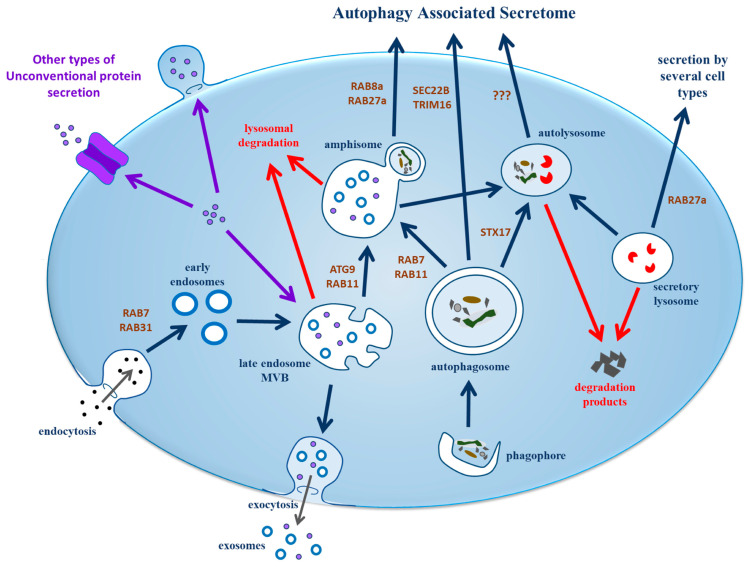
A schematic illustration of secretory autophagy crosstalk with endo/exocytotic pathways and key regulatory molecules. Exosomes originate from the inward budding of the late endosome membrane to form MVBs. They are either released extracellularly upon exocytosis or degraded in lysosomes. In addition, late endosomes/MVBs can also fuse with autophagosomes to produce amphisomes. In turn, amphisomes can either fuse with lysosomes to degrade their content or merge with the plasma membrane to release their cargo into the extracellular environment. Secretory autolysome forms from complex fusion events between autophagosomes, multivesicular bodies, and lysosomes; however, their secretion mechanism remains unclear. Secretory lysosomes are specialized lysosomes containing specific cargo unique to particular cell types, that are trafficked to the membrane. Unconventional secretion can occur via four different pathways, involving either membrane-derived vesicles (MVBs) or by direct translocation through surface transporters or membrane blebbing. The combination of these secretion pathways results in the unique autophagy-dependent secretome. The red arrows indicate the degradation pathway, the blue arrows indicate pathways leading to organelle maturation and to the intersection between autophagic and endocytic pathway fusion with lysosome, and the purple arrows indicate unconventional protein secretion routes. Purple dots represent cargoes trapped within vesicles. Red dots are cargoes that are destined for degradation. “???”—The process of autolysosome secretion is not well-studied, therefore it cannot be described with certainty. The image was created using Microsoft Office PowerPoint software. Version 16.61.

The complex interplay between secretory autophagy and endo/exocytotic pathways, along with the formation, maturation, and secretion mechanisms of various cellular vesicles, is schematically depicted in Figure 1.

## 3. Cross-Talk between Exosomes and Autophagy

### 3.1. Exosomes Biogenesis

Exosomes, membrane vesicles ranging from 30 to 150 nm, are released by cells into various biological fluids such as blood, saliva, urine, breast milk, and cerebrospinal fluid [33].

They carry a diverse cargo that includes RNA (mRNA, miRNA, long noncoding RNA, piwi-interacting RNA, ribosomal RNA, and fragments of t-RNA), DNA, proteins, and lipids from their parent cells. Recognizable exosome markers like CD9, CD63, CD81, Alix, flotillin, TSG101, MHC, HSP70, HSP90, and CD47 are key in their biogenesis, content organization, and secretion [34].

Initially considered mere cellular waste disposals, exosomes are now recognized for their intricate role in cell-to-cell communication, impacting both physiological and pathological processes and serving as potential disease biomarkers. In medicine, exosomes are gaining research interest for their therapeutic potential in treating a variety of diseases as biomarkers and as carriers for gene editing tools, as evidenced by a number of reviews on this topic [35,36].

Exosomes originate within the cell through endocytosis [34]. They are formed by the late endosomal limiting membrane invaginations into small intraluminal vesicles (ILVs), which mature into late endosomes/multivesicular bodies (MVBs). ILVs are ultimately secreted as exosomes through the MVB fusion to the plasma membrane and exocytosis [34]. This process is orchestrated by the ESCRT (Endosomal Sorting Complexes Required for Transport)-dependent mechanism, which selectively includes ubiquitinylated “cargo” components.

In addition to the ESCRT-dependent pathway, exosome biogenesis can also occur via an ESCRT-independent mechanism, which involves various proteins and lipids, including CD63 and ceramides [37,38].

The intracellular trafficking of MVBs, both toward lysosomes for degradation and toward the plasma membrane for exosome release, is orchestrated by various members of the Rab–GTPase family, which also have roles in the regulation of autophagy. For instance, Rab5A is a component of the autophagy initiation complex, which activates VPS34 to generate PI3P, a lipid molecule involved in autophagosome formation [39]. Rab5A also plays a role in the formation of endocytic vesicles and their fusion with early endosomes [40]. In the degradation pathway, Rab7 is responsible for transporting MVBs to lysosomes, where the cargo is degraded, helping regulate cellular energy balance [41]. Rab11, on the other hand, promotes the fusion of MVBs with autophagosomes, leading to the formation of amphisomes, which can be involved in autophagic processes. Furthermore, Rab11 has been reported to act as a platform for ATG proteins during the assembly of autophagosomes [42]. Finally, Rab11, Rab27, and Rab35 participate in the fusion of MVBs with the plasma membrane, facilitating the release of exosomes into the extracellular environment [43].

Apart from Rab GTPases, other protein complexes that regulate vesicular traffic, including SNARE (Soluble N-ethylmaleimide-sensitive factor Attachment protein Receptor) and ESCRT (Endosomal Sorting Complexes Required for Transport), are also shared with autophagy [44]. SNARE complexes, which facilitate membrane fusion events, play pivotal roles in both processes, mediating not only the fusion of MVBs with the plasma membrane for exosome release but also membrane fusion during autophagy. Maturation of autophagosome requires membrane fusion mediated by SNARE family proteins such as VAMP7, syntaxin 7, and syntaxin 8. VAMP7 plays a pivotal role in exosome secretion and is a key molecule for autophagy flux. Thus, SNARE activity could represent the interaction between autophagosome/exosome biogenesis [45,46].

ESCRT’s main function is to form intraluminal vesicles permitting internalization of cytosolic components or membrane-embedded cargoes and promoting endosome maturation. Also, the ESCRT machinery participates in the formation of autophagic vesicles, amphisomes, and the sorting of cargo, demonstrating the convergence of regulatory mechanisms between MVB trafficking and autophagy [47].

The ESCRT-associated protein ALIX plays a crucial role in various membrane processes, including the formation of intralumenal vesicles within multivesicular bodies, exosome release, and viral budding [48]. ALIX interacts with exosomal cargo, suggesting its involvement in cargo packaging and vesicle formation [49,50]. Inhibition of ALIX has been linked to a reduction in autophagy, highlighting the interconnectedness between exosome biogenesis and the autophagy pathway [48].

Exosome biogenesis shares common protein complexes with autophagy, such as SNARE and ESCRT, emphasizing the interconnectedness of these cellular processes. The coordination of these pathways helps maintain intracellular homeostasis and is affected by specific key molecules.

### 3.2. Modulation of Autophagic Processes by Exosomes

The relationship between exosomes and autophagy is increasingly supported by research showing that autophagic molecules are involved in late endosome distribution and exosome biogenesis, and endosomal trafficking components have also been linked to autophagosome maturation. MVBs and exosomes are directed towards autophagic intermediates as autophagy intensifies [41]. However, the regulatory mechanisms overseeing the interactions between exosomes, MVBs, and secreted autophagosomes remain poorly understood.

Exosomes, when internalized by target cells through the endocytic pathway, can impact intracellular activities, including autophagy. There is evidence of a significant connection between exosomes and the regulation of autophagy flux. According to a review by H. Xing and colleagues in 2020 [51], exosomes carry a range of microRNAs (miRNAs) that have been shown to regulate autophagy signaling. These miRNAs, which are small noncoding RNAs, can target and cleave RNAs associated with autophagy. They specifically influence components of the mTOR pathway, Rab GTPases, and ATG factors, which are integral to the autophagy signaling pathway [51]. For example, miR-425-3p facilitated autophagic activation in the recipient cells by targeting AKT1 [52], whereas exosomal miR-190b suppresses autophagy by targeting Atg7 [53]. Additionally, exosome-carried miR-30a can suppress myocardial apoptosis by reducing autophagy via ULK1 and Beclin-1 inhibition [54].

Exosomes can potentially modulate autophagy within target cells through the constituents of their membranes. This modulation may occur either directly through the interactions of these components with cellular machinery or indirectly through a series of intermediary events triggered by the exosomal membrane constituents.

Specifically, the transmembrane protein CD9 has been shown to modulate mitochondrial activity and suppress mitophagy, thereby affecting cellular energy balance. Inhibition of CD9, by a cytopermeable peptide, disrupts the endolysosomal system, reducing early endosomes and amphisomes while increasing lysosomes to preserve proteolysis. However, this leads to impaired mitochondrial turnover and quality. Long-term CD9 depletion can paradoxically augment total mitochondrial mass and restore function. Additionally, cells may upregulate other tetraspanins to compensate and maintain cytosolic component secretion [55].

The tumor susceptibility gene 101 (TSG101), a member of the endosomal sorting complexes, plays a role in the autophagy pathway, particularly in response to rapamycin exposure, which is known to induce autophagy. In neuronal cells, the deletion of TSG101 hampers the autophagic process, evidenced by reduced turnover of MAP1LC3-II, diminished colocalization of Rab7 and MAP1LC3, and decreased cell viability, along with increased levels of p62 and ubiquitinated proteins. This suggests TSG101’s involvement in amphisome formation, promoting autophagic flux upon rapamycin exposure [56]. Furthermore, mice overexpressing TSG101 exhibited improved survival, enhanced cardiac function, reduced inflammation, and activated mitophagy following the administration of endotoxin, in comparison with their wild-type and TSG101-deficient counterparts. TSG101 facilitates the translocation of Parkin to mitochondria, indicating its protective role in cellular stress responses [57].

The CD47 protein, known as integrin-associated protein (IAP), also interacts with thrombospondin-1 (TSP-1) and signal-regulatory protein alpha (SIRPα) [58]. Its role in modulating autophagy varies depending on the cell type. In kidney tissue and in renal tubular epithelial cells (RTECs), CD47 ablation prevents autophagy genes’ (ATG5, ATG7, Beclin1) downregulation under hypoxic stress. The inhibitory action of CD47 in kidney tissue is mediated via CD47-c-myc coupling [59]. On the other hand, CD47 was identified as a critical mediator of proliferation and autophagy to maintain and control mesenchymal stem cell senescence [60]. Additionally, CD47’s autophagy-inducing effect in heart tissue may operate through its coupling with thrombospondin-1 [61].

Exosomes can influence autophagy within recipient cells by delivering autophagic proteins like SQSTM1 and LC3, or their corresponding mRNAs [62,63], which can directly trigger the autophagic process. It is interesting to note that LC3 also plays a role in exosome formation, participating in cargo selection and loading. LC3 was found at the MVB limiting membrane and in intraluminal vesicles (ILVs), where it mediates cargo selection through LC3-interacting regions, ensuring the efficient packaging of cargo into ILVs within MVBs [64].

Exosomes are also known to affect key cellular pathways such as PI3K/Akt/mTOR and AMPK/mTOR, which are crucial for cell survival, by either dampening excessive autophagy or promoting it [65].

Toll-like receptors (TLRs) are critical components of the innate immune system, playing a central role in the recognition of various pathogens, including bacteria, viruses, and fungi. They are known to be involved in the regulation of autophagy [66,67]. TLR2/6 is a heterodimeric receptor that, upon binding with lipoproteins, triggers a signaling pathway resulting in the induction of autophagy. Alvarez-Jimenez et al. demonstrated in their study [68] that exosomes from neutrophils infected with Mycobacterium tuberculosis contain and transfer TLR2/6 ligands, inducing autophagy flux in macrophages.

In conclusion, the intricate relationship between exosomes and autophagy has emerged as a fascinating area of study in recent research. Exosomes have been found to exert a significant influence over various aspects of the autophagic process. This impact is mediated by the delivery of microRNAs, autophagic proteins, their corresponding mRNAs, and even TLR-ligands within exosomes, as well as the transfer of membrane components like LC3, CD9, and CD47.

### 3.3. The Impact of Autophagy on Exosome Maturation and Release

The autophagy machinery significantly influences exosome maturation. Activation of mTORC1 suppresses exosome release, resulting in the accumulation of CD63-positive exosome precursors. Conversely, inhibition of mTORC1, either through rapamycin treatment or nutrient deprivation, enhances exosome release—a process that occurs concurrently with autophagy activation [69]. Another study demonstrates that mTORC-mediated increases in both exosome release and autophagy occur under stress conditions induced by track-etched membrane-based nanoelectroporation (TM-nanoEP) in adipose-derived MSCs. Under these conditions, the negative regulation of mTORC1 correlates with the activation of autophagy and an increase in exosome production. Furthermore, treatment with rapamycin also led to a significant increase in exosome release following TM-nanoEP [70].

Moreover, other stressors, including hypoxia and endoplasmic reticulum stress, have been found to simultaneously raise autophagy flux and exosome secretion in cancer cells, suggesting a concerted response to stress. Specifically, ER stress, known to upregulate autophagy in n various cell types, has been shown to increase MVB formation and exosome release in HeLa cells. Moreover, mitochondrial damage has been linked to an upregulation of autophagy and increased exosome release, which seems to be part of a collective stress response in breast and prostate cancer cell lines (for a review, see Xu et al. [8]). In pancreatic cancer cells, the GAIP-interacting protein C-terminus (GIPC) has been identified as a regulator that simultaneously controls autophagy and exosome production. Knockdown of GIPC results in decreased mTOR activity and increased autophagy flux, along with an increase in exosome production [71].

These observations collectively suggest that the inhibition of the mTOR pathway and various stress conditions simultaneously affect exosome release and autophagy, orchestrating a coordinated strategy to maintain cellular homeostasis and manage waste. It posits that autophagic processes play pleiotropic roles in the regulation of exosome secretion, challenging the traditional view that autophagy primarily contributes to the lysosomal degradation of exosomes. Instead, it appears that autophagy and exosome release might function synergistically as a unified cellular strategy to manage cellular stress.

Accumulating evidence indicates that ATG proteins exert pleiotropic effects on the release of exosomes. Autophagy induction can prevent, while ATG gene silencing or pharmacological inhibition can increase, the extracellular release of exosomes, including those containing pathogenic proteins like α-synuclein [72] and amyloid precursor protein [73]. However, certain ATG genes have been found to stimulate exosome production. The ATG5–ATG16L1 complex, but not ATG7, reduces late endosome acidification by disrupting v-ATPase, hindering subsequent lysosomal degradation and directing them into the secretory pathway rather than the lysosomal pathway [74].

Additionally, cells lacking the ATG12-ATG3 complex, which catalyzes LC3B conjugation, exhibit compromised basal autophagic flux and an accumulation of perinuclear late endosomes. The ATG12-ATG3 complex is also involved in regulating the morphology of MVBs and affects the trafficking of late endosomes, thereby reducing exosome production [48]. Furthermore, the interaction of the ATG12-ATG3 complex with the ESCRT-associated protein Alix (Apoptosis-linked gene 2-interacting protein X, also known as Programmed Cell Death 6 Interacting Protein, PDCD6IP) regulates several Alix/PDCD6IP-dependent processes, including the distribution of late endosomes, biogenesis of exosomes, and virus budding. Alix is essential for efficient basal autophagy, but not starvation-induced autophagy [48].

A small GTPase Rab11 protein associated with MVBs acts as a scaffold for the assembly of ATG proteins in autophagosomes [42].

The scaffold protein GIPC (GAIP interacting protein C terminus) has been identified as a regulator of both autophagy and exosome biogenesis in pancreatic tumor cells. GIPC influences autophagy, likely through metabolic pathways and glucose deprivation, and its knockdown results in increased exosome secretion. Additionally, GIPC not only governs exosome formation but also impacts the content of these extracellular vesicles, highlighting its significant role in coordinating these cellular processes [71].

A novel autophagy secretory pathway has been identified, in which components of the LC3 conjugation machinery determine the incorporation of RNA-binding proteins and small non-coding RNAs into extracellular vesicles, leading to their secretion outside the cell. This process is termed LC3-dependent loading and secretion of EVs (LDELS). Importantly, LDELS differs from classical macroautophagy/autophagy in that it requires components of the LC3 conjugation machinery but no other ATGs involved in autophagosome formation. As EVs have come to act as mediators of intracellular communication, these results provide new insights into how the autophagy mechanism may affect non-cell autonomous cell-to-cell communication [75].

Disruption of lysosomes by bafilomycin A1, as well as genetic inhibition of autophagosome–lysosome fusion, promotes ATG-dependent secretion of autophagy cargo receptors via extracellular vesicular particles, in parallel to autophagy flux inhibition and modulation of EVP secretion, whereby, LC3 and autophagy cargo receptors are secreted as extracellular vesicle-associated proteins, with the obligatory requirement of autophagosome formation. This secretory autophagy requires Rab27 GTPase to occur [76].

This compilation of studies elucidates a complex interrelationship between autophagy and exosome regulation, underscoring the nuanced roles of ATG proteins in exosome maturation and release. The mTORC1 pathway emerges as a critical regulatory axis, modulating exosome release alongside autophagy induction.

Such findings are a prerequisite for understanding the co-existence and mutual regulation of exosome formation and the autophagy process in a single cell characterized as an amphisoma [77].

### 3.4. Biogenesis of Amphisomes

Amphisomes provide a crossroad for the intersection of autophagic and endocytic pathways. Historically defined as degradative compartments, amphisomes originate from the fusion of autophagosomes with multivesicular bodies, the late endosomes, to create hybrid organelles [78]. These can subsequently merge with lysosomes for content degradation or be secreted into the extracellular milieu [78,79,80]. Owing to their dual lineage, amphisomes display markers from both autophagosomes and endosomes, including lipidated LC3 and small Ras-related GTPases such as Rab5, Rab7, and Rab11 [46], alongside V-ATPase [81]. Several key molecules are identified that are involved in amphisome formation, analogous to exosomes—SNARE, Rab GTPases, and ESCRT.

SNARE is a group of proteins (with about 60 representatives) that perform the fusion of intracellular transport vesicles with the cell membrane. These proteins are divided into vesicular proteins (v-SNAREs) and host organelle proteins (t-SNAREs); a new classification subdivides this group into R (arginine)-SNAREs and Q (glutamine)-SNAREs.

Syntaxin 17 (STX17) is an autophagosomal Q-SNARE protein that exclusively localizes to the outer membrane of completed autophagosomes and is essential for autophagosome-to-late endosome fusion [82,83]. STX17 is also required for the transition of autophagosomes to amphisomes. STX17 forms the STX17/SNAP29 complex, which subsequently binds to VAMP8, localized on endosomal and lysosomal membranes. In neurons, STX17 mediates autophagosome fusion with late endosomes and amphisome formation. When the expression of STX17 is inhibited in primary neurons, there is a significant decrease in the number of amphisomes, which coincides with an abnormal accumulation of autophagosomes [84].

In studies on K562 reticulocytes, a human blood cell line, VAMP3/Cellubrevin and VAMP7 have been shown to be other R-SNAREs that control the fusion of autophagosomes and late endosomes to form amphisomes [46].

Rab GTPases are pivotal in the biogenesis of exosomes and also play a crucial role in autophagy. Extensive research has highlighted the involvement of Rab GTPases in the maturation of autophagosomes and their fusion with late endosomes and lysosomes. Furthermore, the Homotypic Fusion and Protein Sorting (HOPS) complex, containing Rab7 GTPase, has been shown to engage with the lysosome-related organelles complex 1 (BLOC-1)-related complex (BORC). This interaction facilitates the assembling of the syntaxin 17/VAMP8/SNAP29 complex, a critical component in amphisome formation. This evidence indirectly points to Rab GTPases playing a role in the genesis of amphisomes. However, the precise mechanisms by which Rab GTPases orchestrate this process are yet to be fully elucidated [85].

Another protein in this family of GTPases that enables amphisome formation is Rab11. Rab11 facilitates endosome–autophagosome fusion by removing Hook, a negative regulator of endosome maturation, from mature late endosomes and inhibiting its homodimerization. Loss of Rab11 leads to the accumulation of abnormal autophagosomes and acidic late endosomes, which is likely a consequence of impaired amphisome formation [86].

Interestingly, constitutively active IKKβ (CA-IKKβ) can induce amphisome formation via decreasing RAB7 expression at the transcriptional level in cancer cells, thereby weakening the lysosomal-dependent degradation pathway. Furthermore, CA-IKKβ activates SNAP23 phosphorylation at the Ser95 site, subsequently modulating the regulation of membrane fusion and the release of small extracellular vesicles (sEVs). This mechanism is believed to be a strategy employed by tumor cells to ensure their survival under stressful conditions [87].

The ESCRT was found to be involved in the regulation of amphisome and lysosome fusion [88].

Cells with mutations in vps28 (ESCRT-I), vps25 (ESCRT-II), or vps32 (ESCRT-III) display an increase in autophagosomes, while the number of amphisomes or autolysosomes is decreased. Additionally, deficiencies in the maturation of autophagosomes and their fusion with lysosomes have been observed in HeLa cells and MEFs depleted of ESCRT-0 VPS27/HRS [89].

Furthermore, the ESCRT-I component TSG101 has been implicated in the regulation of amphisome–lysosome fusion events [90]. The ESCRT-0 component TOM1 is also reported to facilitate the formation of amphisomes through its interactions with myosin VI and autophagy adaptors such as NDP52, T6BF, and optineurin. This suggests that myosin VI may connect TOM1-associated endosomes with autophagosomes, thereby promoting fusion events between autophagosomes and endosomes as part of autophagosome maturation [91].

Collectively, these findings indicate that ESCRTs play a crucial role in autophagosome maturation. They may facilitate the fusion of endosomes and autophagosomes to form amphisomes, thereby acting as a regulatory system for the final maturation of both late endosomes/multivesicular bodies (LEs/MVBs) and autophagosomes. Consequently, alterations in LEs/MVBs could lead to defects in autophagosome maturation and subsequent fusion with lysosomes.

### 3.5. Significance of Amphisomes

Autophagy plays an important role in the cell’s defense against pathogens. However, some microorganisms can influence autophagic processes in the host cell by using them to their advantage.

#### 3.5.1. Amphisomes and Viruses

Amphisomes are among the organelles where Dengue virus replication occurs, but their fusion with lysosomes stops virus replication [92]. Varizella Zoster, in addition to being able to accumulate in host cell amphisomes, uses amphisomes for transportation to the cell surface [93], apparently for subsequent release to the outside. Thus, increased autophagic activity of the cell leads to more active virus dissemination. Some picornoviruses use autophagic pathways to release viral particles wrapped in the host membrane, resulting in minimization of the immune response. In particular, enterovirus D-68 (EV-D68) and coxsackie virus B3 (CVB3) required amphisomes for their maturation [94]. Both EV-D68 and CVB3 disrupt autophagosome–lysosome fusion by cleaving SNAP29 with viral protease 3C. Virus-associated amphisomes or autophagosomes are redirected to the cell periphery and result in nonlytic particle release. Prototype foamy virus (PFV) is a complex retrovirus that can persist in a latent state throughout the life of the organism, suppressing the production of type 1 interferon. Stress Granules (SGs) are an immune complex associated with the antiviral immune response and are critical for type I IFN production. The viral structural protein Gag PFV induces amphisome formation and triggers autophagic clearance of stress granules, thereby attenuating type I IFN production [95].

In addition, amphisomes may also be part of the antiviral mechanism. For example, the recently synthesized tetrapeptide PVF-tet accelerates the production of amphisomes from autophagosomes, and these inducible amphisomes can act as organelle-based antiviral machinery by sequestering the hemagglutinin of the influenza A virus [96]. ORF3a COVID-19 SARS-CoV-2 inhibits autophagy by blocking autophagosome/amphisome fusion with lysosomes. ORF3a localizes to late endosomes and interacts with and sequesters the HOPS component VPS39, thereby preventing the HOPS complex from interacting with STX17 localized in autophagosomes. Consequently, assembly of the STX17/SNAP29/VAMP8 complex is blocked. These results imply that disrupting the interaction between ORF3a and HOPS may represent a potential therapeutic strategy for the treatment of COVID-19 [97].

#### 3.5.2. Amphisomes and Parasites

Parasites of the genus Plasmodia in their highly replicative stage utilize amphisomes of hepatocytes as a source of nutrients. The blockade of autophagy processes reduces the size of plasmodia [98]. Amphisomes are involved in the degradation of intracellular parasites such as chlamydia and the presentation of its antigens by dendritic cells. Chlamydia-containing autophagosomes fuse with MHC I-containing endosomes. In these amphisomes, pathogenic proteins are cleaved to large peptides via cathepsins. These are then cleaved by the proteasome and other components of the classical MHC I pathway. Mature antigens can finally be imported back into the amphisome for loading into MHC I [99].

#### 3.5.3. Amphisome Accumulation

It is known that a mutation in the phosphatidylinositol phosphate kinase (PIPK) domain of the PIKFYVE gene can lead to congenital cataracts resulting from amphisome accumulation in the crystalline lens. Baf-A1, being a specific inhibitor of V-ATPase, leads to a decrease in the amount of amphisomes, suggesting that V-ATPase may be a key component for amphisome formation, and Baf-A1 may be a therapeutic agent that changes the cataract phenotype directly [100].

Amphisome accumulation caused by decreased lysosome efficiency may be a symptom of dysfunctional autophagy, contributing to age-related deterioration of oocyte quality. Amphisome accumulation larger than 10 μm^2^ was observed in aging oocytes, and the same accumulation was found in oocytes treated with the lysosome inhibitor chloroquine. Thus, new prophylactic drugs may be aimed at preventing the decline of lysosomes during oocyte aging, thus maintaining oocyte quality [101].

#### 3.5.4. Amphisomes and the Nervous System

Macroautophagy is required for the continuous remodeling of myelin sheaths, and the complex structure of myelin, with its unique cytosolic and membrane components, requires the coordination of autophagy and endocytosis for its degradation. Aber et al. [102] proposed a model, whereby aggregated myelin basic proteins are taken up in a p62- and Alfy-dependent manner into autophagosomes via selective autophagy. These autophagosomes fuse with endocytosed integral membrane myelin proteins to form an amphisome. The amphisome then fuses with the lysosome, and the contents undergo degradation [102].

In the CNS, amphisomes are required for the degradation of C99B, C83, and amyloid precursor protein (APP) [103], proteins whose accumulation is seen in Alzheimer’s Disease (AD). Along with this, their degradation requires adequate transport by amphisomes to the neuron body. Thus, retrograde transport mediated by the Dynein–SNAPIN complex plays a key role in maintaining axonal homeostasis by promoting autophagic clearance in neurons: amphisomes formed at the distal end of the axon move to the soma where they fuse with lysosomes [104]. In AD, Aβ42 oligomers competitively interact with dynein [105], leading to amphisome accumulation and impairing autophagic clearance. Thus, retrograde transport links may be considered as new point of application in the treatment of AD. It is worth noting that APP is involved in many aspects of neurogenesis and neuronal differentiation [106], and during embryonic neurogenesis, amphisomes are involved in the transport of APP across the neuron and possibly participate in protein processing [107]. Increasing evidence suggests that amphisomes are involved in the autophagy-dependent secretion of tau protein. Induction of autophagy by oxygen–glucose deprivation has been shown to promote the release of free tau protein along with tau in LC3-containing microvesicles, indicating that tau secretion via amphisomes is possible [108].

Parkinson’s Disease (PD) is the second most common neurodegenerative disease and is characterized by the formation of Levi’s Tissues containing α-synuclein [109,110]. Recently, α-synuclein has been shown to be released and transported through amphisome-like structures upon inhibition of the autophagic-lysosomal pathway, suggesting that amphisomes may be alternative organelles for α-synuclein secretion [111].

#### 3.5.5. Amphisomes and Myocardium

Intracellular vesicular dynamics hold a pivotal role in myocardial function, with a primary focus on mitochondria, the central providers of energy in the myocardium. Ensuring the quality control of mitochondria is crucial for maintaining cardiac homeostasis. Furthermore, research has confirmed the presence of various vesicular structures, including exosomes, multivesicular bodies, autophagosomes, and amphisomes, within cardiomyocytes [112,113].

In a healthy myocardium, the elimination of dysfunctional mitochondria is facilitated by intracellular degradation pathways that ultimately converge on the lysosome. One such pathway, macroautophagy, is responsible for the formation of phagophores, autophagosomes, and autolysosomes, playing diverse roles in cardiac viability. During ischemia, autophagy is stimulated through an AMPK-dependent mechanism, and it can serve a protective function. However, when ischemia is followed by reperfusion, autophagy induction occurs in a Beclin 1-dependent manner, resulting in detrimental effects on myocardial function [114]. Additionally, the absence of autophagy has been associated with myocardial dilatation under conditions of pressure overload [115]. Furthermore, in failing myocardium subjected to pressure overload, a decrease in the expression of key autophagic markers such as Beclin1, ATG5, and LC3 has been observed, indicating a downregulation of autophagic flow [116].

Autophagy-related factors play a crucial role in maintaining mitochondrial health, as evidenced by their upregulation in response to targeted mitochondrial damage. This upregulation leads to several beneficial outcomes, including improved mitochondrial membrane potential, enhanced ATP production, and anti-apoptotic effects. These findings strongly suggest the active participation of autophagy in mitochondrial quality control, highlighting its role in preserving the overall health and function of the mitochondria [117].

The concept of secretory autophagy plays a pivotal role in bridging the gap between macroautophagy and extracellular vesicle function, establishing a synergistic and complementary relationship between these processes in the regulation of intracellular protein degradation. Specifically, cardiomyocytes exhibit the remarkable ability to release dysfunctional mitochondria along with other cellular components through a newly identified class of extracellular vesicles referred to as exophers. These exophers are subsequently recognized and taken up by resident macrophages via specific receptors. This intricate process is instrumental in preventing the accumulation of waste products, thereby actively contributing to the preservation of cardiomyocyte function and overall cardiac health [118].

These findings find further support in recent research conducted on HeLa cells, where authors have also elucidated the mechanisms underlying the removal of damaged mitochondria from cells through the ATG8-dependent secretory autophagy pathway [25]. This emerging understanding highlights the conserved nature of these critical cellular mechanisms across different cell types and reinforces their significance in maintaining cellular and organ health.

The study conducted by Wenjing Liang et al. [119] uncovers an intriguing alternative mechanism for the elimination of mitochondria in situations where lysosomal function is compromised. Their research demonstrates that inhibiting lysosomal degradation, achieved by deleting the small GTPase Rab7 in cells or within the adult mouse heart, leads to a notable increase in the secretion of EVs containing ubiquitinated cargos, including intact mitochondria. Furthermore, heart samples from both aged mice and individuals with Danon disease exhibited elevated levels of secreted EVs containing mitochondria, indicating the activation of vesicular release mechanisms in these contexts.

Conversely, when researchers explored the consequences of increasing the number of intracellular autophagic structures in hearts overexpressing Atg7, along with inhibiting lysosomal acidification using chloroquine, they observed a notable increase in the number of amphisomes. This increase in amphisome numbers resulted from the autophagosomal engulfment of cargo and their subsequent fusion with vesicular endosomes [113]. These findings underscore the dynamic nature of cellular quality control mechanisms, revealing alternative pathways that come into play when traditional lysosomal degradation is compromised, further highlighting the adaptability of cells in maintaining their homeostasis.

The study conducted by Yang Y et al. [120] provides valuable insight into the intricate interplay between exosomal miR-30a, apoptosis, and autophagy in cardiomyocytes under hypoxic conditions. Their findings reveal that inhibiting exosomal miR-30a can have a significant impact on the apoptosis of cardiomyocytes induced by hypoxia. This effect is achieved through the augmentation of autophagy, as evidenced by the enhanced expression of core autophagy regulators such as beclin-1, Atg12, and LC3Ⅱ/Ⅰ.

#### 3.5.6. Amphisomes and Exosomes

Antagonistic interactions between autophagy and exosome release, often in the form of amphisome degradation, have been well-documented. For instance, under conditions of starvation or rapamycin treatment in the erythroleukemic cell line K562, autophagy induction is accompanied by enhanced fusion of autophagosomes with multivesicular bodies (MVBs), leading to a reduction in exosome release [41].

Indeed, this phenomenon suggests that reduced exosome release may serve as a cellular strategy to redirect MVBs towards degradation via the autophagic pathway, potentially facilitating the recycling of MVBs for energy production. Furthermore, in studies involving mammalian cell lines and mouse models, a noteworthy observation has been the conjugation of the ubiquitin-like protein ISG15 with TSG101, a critical component of the ESCRT-I complex. This molecular event has been found to promote several significant cellular outcomes, including protein aggregation, degradation, reduced MVB numbers, and a consequential decrease in exosome release [121]. These findings underscore the intricate regulatory mechanisms that govern the balance between exosome secretion and intracellular degradation processes, ultimately influencing cellular homeostasis and intercellular communication.

The prevention of endosome–lysosome fusion, achieved either through bafilomycin A1 treatment or by inhibiting autophagy, consistently results in a significant reduction in exosome release. This underscores the active role of autophagy in the lysosomal degradation of MVBs [121]. Collectively, these studies highlight the widespread occurrence and importance of autophagic processes in the degradation of MVBs, emphasizing the complex relationship between autophagy and exosome-related pathways within the cellular context.

Furthermore, emerging research has identified the role of Cav1 in autophagy regulation, particularly concerning cholesterol-dependent modulation of Cav1 trafficking and its impact on amphisome biogenesis [122]. Cav-1 is localized in the plasma membrane of melanoma cells, where it forms functional caveolar structures. Interestingly, Cav-1 plays a dual role in cellular processes related to MVBs and exosome secretion. In this context, the presence of Cav-1 is associated with the inhibition of autophagy. However, an intriguing shift occurs when cholesterol transport in melanoma cells is disrupted. Under these conditions, Cav-1 undergoes a relocation, moving from the plasma membrane to the endolysosomal compartment. This relocation of Cav-1 significantly enhances the autophagy flux, which leads to the formation of amphisomes and amphisome-dependent secretion of sEVs, also known as small exovesicles [122].

Additionally, studies in mouse intestinal goblet cells have revealed that LC3B co-localizes with endosomal markers, such as EEA1, RAB7, and RAB11, on amphisome-like organelles. These organelles play a crucial role in the production of reactive oxygen species, which, in turn, regulate mucin granule secretion [123]. Similarly, amphisomes in lung epithelial cells serve secretory functions and act as intermediates in processes like interferon-γ (IFN-γ)-induced secretion of annexin A2 [19].

**Figure 2:**
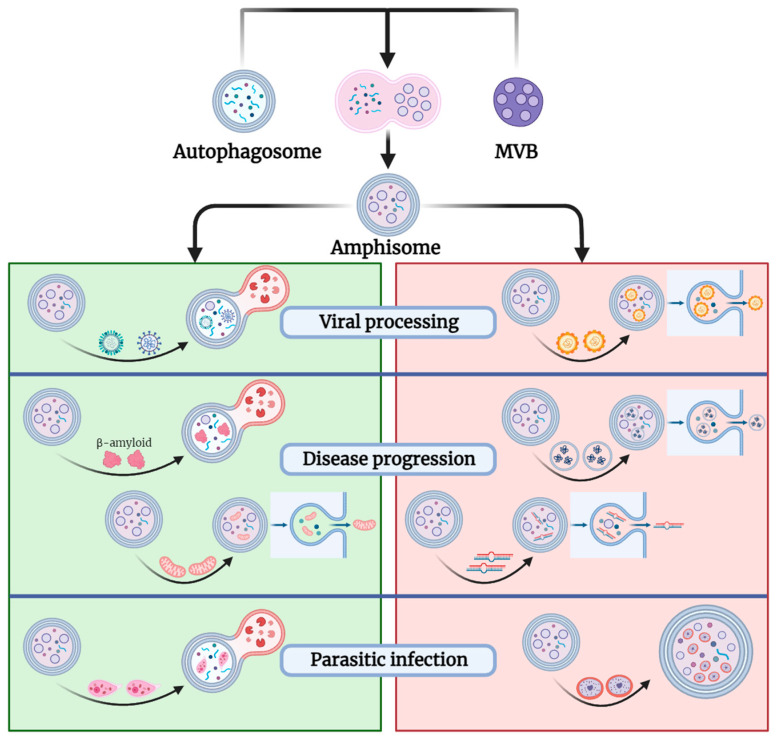
Dual role of amphisomes in various pathological states. The fusion of the autophagosome with the multivesicular body results in the formation of amphsiomes, which may further engulf distinct intracellular particles and finally fuse with the lysosome or cellular membrane to digest or release its content, respectively. For example, amphisomes have the capacity to capture influenza and COVID-19 viruses, β-amyloid, and *Chlamydia* spp. for their subsequent degradation through fusion with lysosomes or facilitate the removal of dysfunctional mitochondria by exocytosis. In contrast, the Varicella Zoster virus, plasmodia, as well as the amyloid precursor protein, exploit amphisomes to bypass the host defense system and disseminate throughout tissues. Meanwhile, the extracellular release of miR-30a via amphisomes contributes to disease progression. The image represents the fine and intricate architecture of the amphsiome signaling system, where the green box reflects the beneficial impact of amphsiomes, whereas the red box—detrimental. The illustration was created with BioRender.com (accessed on 28 December 2023).

Altogether, these findings highlight the intricate interplay between autophagy and exosome-related pathways, with each pathway capable of compensating for the deficiencies of the other, thereby functioning alternatively. Figure 2 illustrates the dual role of amphisomes in various pathological states, underscoring how these structures can either contribute to cellular defense mechanisms or be co-opted by pathogens to further disease progression.

## 4. Authophagy-Dependent Secretion

Autophagy-dependent secretion is often induced by cellular stress, which can encompass a wide range of stressors, including redox stress, and various environmental factors. These stressors have the capacity to activate autophagy, leading to the secretion of proteins through this unique pathway. Cellular stress triggers a cascade of events, activating various processes such as lysosome exocytosis, exosome formation, membrane vesiculation, autophagy, and even pyroptosis. These processes can be interconnected, contributing to autophagy-dependent secretion [124,125].

Secretory autophagy, a recently identified pathway, differs from traditional autophagy as it involves autophagosomes fusing with the plasma membrane instead of lysosomes. A significant body of research has associated secretory autophagy with immune responses and inflammation. However, a fundamental question remains unanswered: is the primary role of secretory autophagy to eliminate proteins or to repurpose them for extracellular functions?

The list of proteins secreted via secretory autophagy continues to grow, encompassing a diverse array of leaderless secretory proteins (LLSPs), including some of the most well-known LLSP proteins, including IL1b, IL6, IL8, IL18 [126], and HMGB1 [127], which have been demonstrated to rely on autophagic machinery for their unconventional secretion. Notably, a shared characteristic of LLSP pathways is their inducibility by stress, further underscoring the role of stress in modulating this process.

Secretory autophagy, however, extends beyond inflammation-associated cytokines, with various LLSP proteins making appearances in the autophagy-dependent secretome. These proteins encompass diverse categories, including galectins (e.g., galectin-3), cytoskeletal proteins like tubulin, and annexin-I [128]. Moreover, secretory autophagy has been linked to the secretion of hormones like adiponectin [129] and proteolysis regulators like TIMP1 [130], MMP2 [25], MMP9 [30], and even entire organelles such as mitochondria [31]. Remarkably, stress induction also plays a pivotal role in promoting the secretion of most of these substances.

Another noteworthy protein released via secretory autophagy is the anti-inflammatory cytokine TGF-β1. This secretion follows an unconventional pathway that relies on autophagic machinery and cytoskeletal regulators, as elucidated by Nüchel J. et al. [20]. Furthermore, proteins like insulin-degrading enzyme (IDE) [131] and STING [32] have recently emerged as substrates of secretory autophagy in response to stress.

In a comprehensive investigation [30] involving proteomic secretome-wide analysis, the examination of the secretome from an autophagy-deficient microglial cell line (Atg5 KO) under stress conditions induced by dexamethasone yielded intriguing results. Specifically, it was observed that the secretion of 530 proteins out of a total of 710 (comprising 75%) was significantly enhanced. This discovery unequivocally confirms the existence of an autophagy-regulated secretome. Notably, the functional profile of this autophagy-regulated secretome includes a repertoire of crucial proteins, such as vimentin (vim), matrix metalloproteinase 9 (mmp9), chemokine ligand 2 (ccl2), insulin-like growth factor 1 (igf1), and heme oxygenase 1 (hmox1), all of which play pivotal roles in immune responses.

The possibility of an autophagy-dependent secretome in vivo warrants further investigation and may lead to the identification of proteomic signatures of autophagy activation as clinically useful serum biomarkers. Theoretically, autophagy-inducing therapies might lead to untoward effects via the unconventional secretion of pro-inflammatory mediators or other pathogenic proteins. Mechanisms and peculiarities of the secretion of some of these aforementioned factors will be discussed in detail below.

Interleukin-1β (IL-1β) secreted upon various stress stimuli is a key pro-inflammatory cytokine. It stimulates neutrophilia and acute phase proteins, enhances the proliferation of B and T lymphocytes to antigens/mitogens, and induces the production of proteolytic enzymes and prostaglandins by fibroblasts, chondrocytes, and a number of other cells. In addition, it upregulates the expression and production of chemokines (IL-8, MCP-1) and inflammatory cytokines (TNFα, IL-6, IL-1), induces the production of ROS and NO, causes redistribution of zinc and iron in the tissues, regulates corticosteroids and glucose homeostasis, and is a potent adjuvant of antigen-specific antibody responses in vivo [132]. Zhang and colleagues showed that cytosolic IL-1β is able to associate with HSP90, which facilitates the translocation of IL-1β into the lumen of a vesicle carrier that later either transforms into or fuses with a phagophore and an autophagosome. IL-1β localizes to a space between the outer and inner membranes after the formation of the double-membrane autophagosome. This topological distribution ensures the secretion of IL-1β in a soluble form. The IL-1β-containing autophagosome can fuse directly with the plasma membrane or fuse with a MVB to form an amphisome and then merge with the plasma membrane [133]. Interleukin-36γ (IL-36γ), a member of the IL-1 superfamily, is a cytokine that plays an important role in pathogen recognition and response, wound healing, and the maintenance of epithelial barriers. IL-36γ dysregulation is implicated in several diseases of the epithelium, including recurrent respiratory papillomatosis and psoriasis. Papayannakos and colleagues have shown that poly(I:C)-induced secretion of IL36γ by human foreskin keratinocytes (HFKs) relies on the mTOR-dependent formation of autophagosomes, promoting, as the authors suggest, stress relief [134]. Moreover, the same research group has shown that IL36γ co-migrates with LC3b-II and markers of multivesicular bodies during subcellular fractionation of homogenates derived from poly(I:C)-stimulated HFKs and may serve as a selective cargo of an autophagosome and/or amphisome, prior to its secretion in small extracellular vesicles [135].

Autophagy-associated proteins, including ATG7 and ATG12, have been implicated in facilitating the production and conventional secretion of the cytokine IL-6. This cytokine plays a crucial role in driving oncogenic RAS invasion of epithelial cells and pulmonary metastasis in vivo. However, it is important to note that while some authors suggest that autophagy promotes cancer progression through the secretion of various factors, including IL-6, these claims lack molecular definition. To gain a more comprehensive understanding of the role of autophagy in unconventional secretion, further evidence is needed [136]. Additionally, studies have shown that blocking autophagy by siRNA knockdown of Beclin-1 can modulate the levels of secreted IL-6, suggesting that autophagy is a contributing factor in the secretion of these cytokines [137].

Annexin A2 p36 (ANXA2), a Ca^2+^-dependent membrane-binding protein, is expressed abundantly in macrophages, monocytes, cancer, endothelial, and epithelial cells. ANXA2 can exist as a monomer or as a heterotetrameric complex with S100A10 (p11). Annexin A2 has a wide range of biological functions depending on its localization in the cell. For instance, surface ANXA2 serves as a receptor for p11 to form a heterotetrameric (ANXA2-p11)2 complex and binds to the plasminogen and tissue plasminogen activator, thereby regulating cell migration, fibrinolysis response, and other cellular functions. Nuclear ANXA2 has been shown to bind RNA and has also been suggested to participate in the primer recognition protein complex that is involved in enhancing DNA polymerase α activity. ANXA2 also takes part in the regulation of the cytoskeletal network, membrane dynamics, endocytosis, and exocytosis [138]. Chen and colleagues demonstrated that IFNγ-stimulated exocytosis of ANXA2 in lung epithelial cells (LECs) occurs through autophagy induction and subsequent formation of ANXA2-containing amphisomes, which do not appear to fuse with lysosomes but rather merge directly with the plasma membrane to release ANXA2. Interestingly, RAB11 is essential for transporting ANXA2 to the amphisome, while RAB8A and RAB27A, but not RAB27B, are involved in the fusion of ANXA2-containing amphisomes with the plasma membrane. This intracellular-to-surface translocation of ANXA2 promotes, at least, the phagocytosis of apoptotic cells by LECs [19]. Therefore, it can be assumed that the autophagic machinery with amphisome formation may serve as a key mechanism in the acquisition of a new secretory phenotype implemented by cells exposed to a number of stressors, such as poly(I:C) and IFNγ.

A non-histone nuclear protein high mobility group box 1 (HMGB1), acts as a DNA chaperone that repairs and stabilizes the chromatin structure. Since HMGB1 acts as a danger-associated molecular pattern, it is secreted by cells in response to various stress stimuli in order to promote sterile inflammation and cell migration. Kim and colleagues elucidated the mechanism of HMGB1 secretion and showed that heat shock protein 90 alpha family class A member 1 (HSP90AA1) enhances HMGB1 nuclear-cytoplasmic translocation, and Golgi reassembly stacking protein 2 (GORASP2) mediates HMGB1-containing cargo trafficking in the cytoplasm. GORASP2 and HSP90AA1 also promote cellular autophagy, and the authors suggest that HMGB1 secretion is regulated by both HSP90AA1- and GORASP2-mediated secretory autophagy machinery, involving autophagosome formation and maturation, but not by autophagosome–lysosome fusion. This is supported by the fact that the knockdown of RAB8A, RAB11A, and RAB27A decreases HMGB1 secretion upon chloroquine, an autophagy inhibitor treatment, but RAB7A knockdown does not. RAB7A is involved in MVB and lysosome fusion, and RAB8A, RAB11A, and RAB27A are known to induce components involved in exosome secretion. HMGB1 secretion is diminished by the early autophagy inhibitor wortmannin, an inhibitor of the phosphatidylinositol 3-kinase, upon either starvation conditions or treatment with phorbol 12-myristate 13-acetate and trichostatin A, activators of HMGB1 secretion. Furthermore, HMGB1 secretion induced by overexpression of HSP90AA1, GORASP2, and ADP ribosylation factor 1 is reduced by treatment with GW4869, a sphingomyelin phosphodiesterase 3 inhibitor used to inhibit ceramide-dependent MVB formation [139]. This suggests that amphisome formation is required for HMGB1 secretion since inhibition of both autophagy and MVB formation leads to a reduction in HMGB1 secretion.

Secretory autophagy is crucial for the secretion of the tissue inhibitor of metalloproteinase 1 (TIMP1), a key regulatory protein in fibrosis that inhibits matrix metalloproteinases (MMPs). TIMP1 also functions as a cytokine-like signaling molecule, influencing a range of biological processes such as cell growth, apoptosis, differentiation, angiogenesis, and oncogenesis [140]. The process of TIMP1 secretion through autophagy is mediated by the fusion of autophagosomes with Rab37, LC3, and Sec22b-anchored vesicles—essential for its release into the extracellular space. The suppression of autophagy-related genes Atg5 or Atg7 has been shown to reduce TIMP1 secretion, highlighting the vital role of autophagy in TIMP1 regulation. These insights into TIMP1′s secretory pathway through autophagy present significant implications for understanding cancer metastasis and potential therapeutic interventions [130].

The E2 subunit of the pyruvate dehydrogenase complex (PDCE2) is localized on the inner mitochondrial membrane and is part of the enzyme complex responsible for the conversion of pyruvate to acetyl-CoA in the mitochondrial respiration process. In damaged and stressed cells, PDCE2 can be translocated from the mitochondria to the cell surface and, in some cases, may act as an autoantigen [141,142]. Using human cerebral microvascular endothelial cells (hCMECs), Faqihi and colleagues sought to investigate the translocation mechanism and showed that sub-lethal doses of ionizing radiation cause hCMECs senescence and late-stage blockage of autophagic flux, thereby reducing lysosomal activity and promoting trafficking of PDCE2-containing autolysosomes to fuse with the plasma membrane. At the same time, PDCE2 co-localized with the SNARE protein SEC22B and the lysosome marker LAMP1, whereas PDCE2 association with the late endosome marker RAB7A was minimal. The authors believe that PDCE2 may become an appropriate target for drugs targeting radiation-damaged vascular endothelium [142].

Members of cytoplasmic phosphoproteins (PACSINs) play an important role in vesicle formation and transport. PACSINs regulate intracellular vesicle trafficking, cytoskeletal rearrangement, caveolar biogenesis, neuronal development, and cell migration [143]. Oe and colleagues demonstrated that PACSIN1 is required for the fusion of autophagosome/amphisome with lysosome since PACSIN1 is indispensable for the assembly of STX17-SNAP29-VAMP8 and YKT6-SNAP29-STX7 SNARE complexes that mediate this fusion. Interestingly, PACSIN1 does not interact with RAB7, an effector of MVB fusion with lysosomes. Moreover, PACSIN1 was found to be required for lysophagy and aggrephagy, but not for mitophagy. The authors suggest that in basal autophagy, characterized by relatively low autophagic activity, the fusion path via amphisomes is preferentially selected, whereas in starvation conditions associated with significantly increased autophagic activity, direct fusion of autophagosomes with lysosomes may be preferred [144].

In their study, Kuramoto et al. identified a role for secretory autophagy in the release of adiponectin, an essential hormone for metabolic regulation, derived from adipose tissue. The research elucidated that Beclin 1 (Becn1) facilitates the secretion of adiponectin by binding to the Sec6 component of the exocyst, a vesicle trafficking complex. This binding is crucial for the docking of adiponectin-laden vesicles to the plasma membrane and their subsequent exocytosis. Intriguingly, the mechanism described by Becn1 for adiponectin release is mechanistically distinct from the unconventional secretory routes utilized by cytokines such as IL-1β and IL-18, which depend on autophagosome-like vesicles. This discovery not only provides insight into adiponectin trafficking but also suggests new regulatory pathways that secretory autophagy may influence, offering potential therapeutic targets for metabolic diseases [129].

MVB-mediated exosomal secretion is known to be an essential regulator of healthy reproduction, protecting high quality spermatozoa and eliminating feeble and defective spermatozoa. These exosomes interact with germ cells to maintain the homeostasis of spermatogenesis. The exosomal cargo proteins act together to ensure proper sperm motility, metabolism, oxidation-reduction, acrosome reaction, capacitation, and fertilization [145]. Ali and colleagues revealed that exposure to detrimental amounts of cadmium leads to multiple defects in the murine reproductive system and hypo-spermatogenesis, characterized by significantly reduced biogenesis of MVBs and exosomes by Sertoli cells and upregulated autophagy with an increased formation of autophagosomes/amphisomes destined for lysosomal degradation [146]. This report suggests that stress stimuli may exert their pathogenic influence not only by exploiting exosomes, enriching them with pro-inflammatory factors, but also by hyperactivating autophagy, causing a dramatic reduction in exosome biogenesis.

S-phase kinase-associated protein 1 (SKP1) is the assembly factor of SKP1–CUL1–F-box protein complexes, also known as CUL1-RING ubiquitin ligases, a family of E3 ligases found in all eukaryotes, which mediate the proteasomal degradation of hundreds of cellular regulatory proteins [147]. Li and colleagues showed that SKP1 plays a key role in the regulation of secretory autophagy. Specifically, under conditions of nutrient starvation, SKP1 is phosphorylated at Thr131, allowing its interaction with the V1 subunits of the vacuolar ATPase (V-ATPase), pumping protons into the lumen of organelles such as lysosomes, the Golgi apparatus, and secretory vesicles, coupled with ATP hydrolysis. This interaction promotes assembly of the V-ATPase and thereby leads to an increase in acidification of late endosomes and MVBs. On the contrary, under nutrient-rich conditions SUMOylation of phosphorylated SKP1 allows its binding to and dephosphorylation by the Mg^2+^/Mn^2+^ Dependent Protein Phosphatase 1B. In turn, unphosphorylated SKP1 interacts with the SNARE protein SEC22B to promote unconventional secretion of amphisome cargo. Therefore, under starvation conditions, SKP1 switches the autophagic machinery from exocytosis of amphisomes to acidification of these hybrid autophagic compartments for their proteasomal degradation [148].

The cGAS-STING pathway plays an important role in innate immunity, serving as a sentinel for detecting cytosolic DNA—an indicator of viral infections or genomic instability. Within this pathway, the cyclic GMP–AMP synthase (cGAS) acts as a DNA sensor. Upon binding to cytosolic DNA, cGAS catalyzes the synthesis of cyclic GMP–AMP, which then binds to the stimulator of interferon genes (STINGs) transmembrane protein residing in the endoplasmic reticulum membrane [149]. This binding triggers a conformational change in STING, leading to its activation. Active STING, in turn, recruits and activates the TANK-binding kinase 1 (TBK1) and the transcription factor interferon regulatory factor 3 (IRF3). This cascade ultimately culminates in the production of type I interferons (IFNs) and various proinflammatory cytokines, setting in motion an antiviral immune response. Intriguingly, recent research by Gao et al. [32] has unveiled that activated STING can be packaged into autophagosomes and subsequently secreted as cargo within a unique type of extracellular vesicle (EV) named R-EV (RAB22A-induced extracellular vesicle). Gao’s study further elucidates the molecular mechanism behind this phenomenon. RAB22A, a small GTPase, binds to PI4K2A, which generates PI4P—a lipid crucial for autophagosome formation. This interaction recruits the Atg12–Atg5–Atg16L1 complex, leading to LC3-II anchoring on ER-derived membranes, thereby forming the RAB22A-mediated non-canonical autophagosome. Activated STING localizes on the membrane of these autophagosomes. Notably, RAB22A also inactivates RAB7, suppressing Rafeesome fusion with lysosomes. As a result, vesicles containing activated STING are secreted as R-EVs, activating the IFNβ pathway in recipient cells within the tumor microenvironment, thereby facilitating antitumor immunity. Additionally, it has been observed that active STING can promote the phosphorylation of ULK1, a key regulator of autophagy initiation, leading to increased autophagic activity. This discovery highlights the bidirectional regulation between the cGAS–STING pathway and autophagy, establishing a feedback loop that fine-tunes immune responses [32].

In conclusion, secretory authophagy is crucial for maintaining a healthy and physiological cellular state, as well as in response to various stress stimuli such as poly(I:C) and IFNγ. Thus, extracellular vesicles play a key role in the immune response and the propagation of inflammation. Several cell types, such as Sertoli cells, exert their functions in part through the extracellular release of these vesicles. Any disruption of secretory autophagy can compromise not only individual cells or tissues but the overall homeostasis of the organism.

The primary role of secretory autophagy—whether it is to dispose of proteins or to repurpose them for extracellular functions—remains a topic of investigation. Secretory autophagy enables the release of numerous biologically active molecules, mainly involved in inflammation regulation, which may prefer this pathway to avoid posttranslational modifications that could affect their activity and function. Such modifications can occur during conventional secretion through the ER–Golgi pathway and include changes such as N-glycosylation or disulfide bond formation. Conversely, it is an intriguing possibility that mechanisms like unconventional protein secretion have evolved to handle proteins that are mutated, overexpressed, or otherwise potentially harmful, especially in cells with limited proteolytic processes. As we continue to unravel the mysteries of secretory autophagy, it becomes increasingly clear that its roles extend beyond simple waste management. It is intimately involved in orchestrating a wide array of cellular events, from stress response to intercellular communication, and it holds substantial promise for novel therapeutic strategies targeting diseases characterized by inflammation and protein mismanagement.

## 5. Conclusions

The dynamic interplay between extracellular vesicles, particularly exosomes, and the autophagic process forms a sophisticated network of intracellular and extracellular interactions. This review sheds light on the nexus of these processes rather than exhaustively covering the experimental study of membrane structures.

The role of exosomes can generally be assessed as mediatory in the transfer of information between cells in the form of biologically active molecules. Apparently, exosomes are the main factor of the so-called “paracrine” influence on neighboring cells and tissues of the organism, which is actively used in clinical practice. Conversely, pathogens may co-opt exosomes to disseminate infectious material.

Autophagy is traditionally associated with the degradation of excess and damaged cellular components, serving as a primary mechanism for maintaining cellular homeostasis. Beyond its role in degradation and quality control of intracellular organelles, autophagy also extends its functions to the regulation of intracellular signaling [150].

Secretory autophagy emerges as a dual-edged sword: a cellular defense against toxic substances and pathogens, yet exploitable by the same under pathological conditions. The dysregulation of autophagy mediators can drastically alter the cellular secretory profile. This affects the secretion of a plethora of factors ranging from cytokines to granule contents, and even viral particles, influencing a broad spectrum of diseases from cancer to neurodegeneration.

Both exosome biogenesis and autophagy, including its secretory form, engage numerous regulatory proteins that extensively interact with cellular factors across various processes. The shared regulatory proteins and pathways, such as mTORC1, highlight the potential for therapeutic targeting of intracellular membrane dynamics to ameliorate pathological states.

Thus, understanding the regulatory mechanisms governing exosome formation and the mutual regulation of extracellular vesicle biogenesis and autophagy is a promising avenue for modulating intracellular mechanisms in disease. The breadth of secretory autophagy’s roles—from cellular cleanup to intercellular communication—underscores its importance in both health and disease, providing a rich landscape for future therapeutic exploration.
